# Mr. Charles Terry

**Published:** 1910-06-04

**Authors:** 


The death is announced of Mr. Charles Terry, at the age of eighty-five. In 1849 Mr. Terry became a M.R.C.S. Eng., and L.S.A. the year following. At one time he held the post of resident medical officer at the Royal Mineral Water Hospital, Bath, but at the time of his death he had retired from practice.

